# Neuroimaging and clinical features of bilateral Wallerian degeneration of middle cerebellar peduncles subsequent to pontine infarction

**DOI:** 10.1111/cns.14828

**Published:** 2024-07-01

**Authors:** Yao Zhang, Ting Wei, Hui Yu, Wenli Li, Wenqian Luo, Bin Liu

**Affiliations:** ^1^ Department of Neurology The First Affiliated Hospital of Shandong First Medical University, Shandong Provincial Qianfoshan Hospital Jinan China; ^2^ Department of Neurology, Shandong Provincial Qianfoshan Hospital, School of Clinical Medicine Weifang Medical University Weifang China; ^3^ Shandong Institute of Neuroimmunology Jinan China

**Keywords:** middle cerebellar peduncles, neuroimaging, pontine infarction, Wallerian degeneration

## Abstract

**Objective:**

Wallerian degeneration (WD) of the middle cerebellar peduncles (MCPs) following pontine infarction is a rare secondary degenerative neurological condition. Due to its infrequency, there is limited research on its characteristics.

**Methods:**

This study aims to present three cases of WD of MCPs following pontine infarction and to analyze the prognosis, clinical manifestations, and neuroimaging features by amalgamating our cases with previously reported ones.

**Results:**

The cohort consisted of 25 cases, comprising 18 men and 7 women aged 29 to 77 years (mean age: 66.2 years). The majority of patients (94%) exhibit risk factors for cerebrovascular disease, with hypertension being the primary risk factor. Magnetic resonance imaging (MRI) can detect WD of MCPs within a range of 21 days to 12 months following pontine infarction. This degeneration is characterized by bilateral symmetric hyperintensities on T2/FLAIR‐weighted images (WI) lesions in the MCPs. Moreover, restricted diffusion, with hyperintensity on diffusion‐weighted imaging (DWI) and low apparent diffusion coefficient (ADC) signal intensity may be observed as early as 21 days after the infarction. Upon detection of WD, it was observed that 20 patients (80%) remained asymptomatic during subsequent clinic visits, while four (16%) experienced a worsening of pre‐existing symptoms.

**Conclusions:**

These findings underscore the importance of neurologists enhancing their understanding of this condition by gaining fresh insights into the neuroimaging characteristics, clinical manifestations, and prognosis of individuals with WD of bilateral MCPs.

## Graphical abstract

Wallerian degeneration (WD) of bilateral middle cerebellar peduncles (MCPs) following pontine infarction is a rare secondary neurodegenerative disorder. WD of bilateral (MCPs) does not seem to be a marker for a bad outcome in general. The radiological features in MRI included hyperintense T2WI signals, hyperintense FLAIR signals, high DWI signal intensity, and low ADC signal intensity.

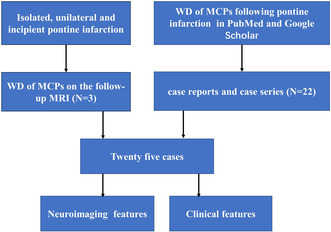

## 1 INTRODUCTION

Wallerian degeneration (WD) is a progressive demyelination and breakdown of distal axons that occurs after neuronal or proximal axonal injury, frequently following stroke.^1^ However, WD can arise due to various factors such as hemorrhage, necrosis, tumors, trauma, focal demyelination, white matter lesions, and multisystem atrophy.^2^ WD can manifest approximately 1 week after a stroke.^3^ Among the brain structures, the corticospinal tract is most susceptible to WD following diverse insults.^4^, ^5^

According to current knowledge, the occurrence of WD in bilateral middle cerebellar peduncles (MCPs) after pontine infarction is exceedingly rare. However, a comprehensive understanding of its characteristics remains limited due to its low incidence. Previous literature primarily comprises case reports and small retrospective clinical studies regarding WD in MCPs following pontine infarction.^4^, ^5^ This study presents three cases demonstrating symmetrical WD in bilateral MCPs after pontine infarction. Furthermore, a literature review explored clinical and radiological features to enhance awareness of this condition. This study aimed to improve the understanding of WD in MCPs secondary to pontine infarctions.

## 2 METHODS AND PATIENTS

### 2.1 Methods

This study received approval from the ethics committee of Shandong Provincial Qianfoshan Hospital and was conducted at a single institution. Data were collected from patients diagnosed with isolated, unilateral, and incipient pontine infarction who were admitted to Shandong Provincial Qianfoshan Hospital between February 2021 and February 2023. Patients with bilateral MCPs demonstrated WD on follow‐up MRI were included in the study. Patients who exhibited low compliance during follow‐up, were excluded. Ultimately, three patients were included as the case group. To conduct a comparative analysis of the radiological and clinical characteristics, a control group of patients with pons infarction, who did not exhibit bilateral MCPs on MRI during a follow‐up period (>1 year), was formed. Finally, a total of 92 patients were enrolled as the control group. Clinical data included gender, age at onset, risk factors for cerebrovascular disease, symptoms, radiological findings, National Institute of Health Stroke Scale (NIHSS) score, follow‐up duration, and outcomes. Conventional MRI scans were performed using a 1.436 T MRI system (uMR586, United Imaging, Shanghai, China) or a 3.0 T MRI system (Discovery MR750, GE, USA). MRI sequences included T1‐weighted imaging (WI), T2WI, fluid‐attenuated inversion recovery (FLAIR), diffusion‐WI (DWI), and apparent diffusion coefficient (ADC) maps.

### 2.2 Literature search

Furthermore, a comprehensive analysis of available literature was performed to elucidate the clinical and radiological characteristics of patients with WD in bilateral MCPs secondary to pontine infarctions. Our survey was restricted to case reports and case series regarding WD in bilateral MCPs post‐pontine infarction. A comprehensive search was conducted in the PubMed and Google Scholar databases using the search terms “Wallerian degeneration AND middle cerebellar peduncles” to identify relevant case descriptions published between January 1, 2001 and December 30, 2022. Non‐peer‐reviewed publications were excluded from our study.

### 2.3 Statistical analysis

Statistical analysis was performed using SPSS statistical software version 17.0 (SPSS Inc., Chicago, IL, USA). Measurement data are presented as mean ± standard deviation; count data are presented as ratio. Intergroup comparison of count data was performed using the Chi‐squared test; a value of *p* < 0.05 was considered statistically significant.

### 2.4 Case group patients

#### 2.4.1 Patient 1

**FIGURE 1:**
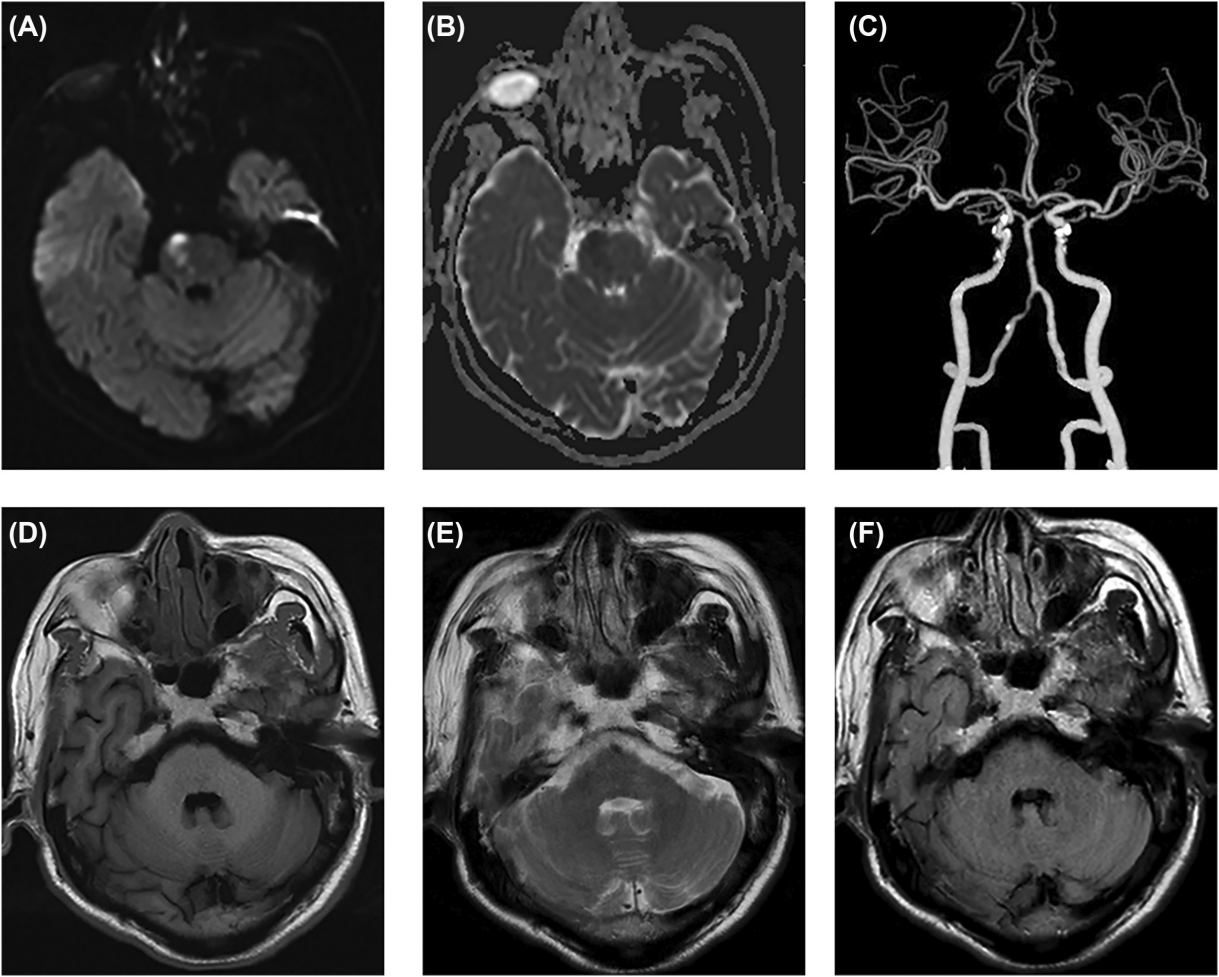
The initial MRI examination of patient 1 revealed the presence of an acute infarction in the right pons. (A, B) exhibited high signal intensity on diffusion‐weighted imaging (DWI), and low signal intensity on the apparent diffusion coefficient (ADC) map, in the right anterior pons. Computed tomography angiography (CTA) demonstrated the presence of multifocal stenosis in both the right vertebral artery (V4 segment) and basilar artery (C). (D) Axial T1 weighted image, (E) axial T2 weighted image and (F) axial FLAIR weighted image did not exhibit any abnormal signal patterns in the bilateral middle cerebellar peduncles.

A 72‐year‐old man with sudden‐onset slurred speech and left limb weakness persisting for 6 h was admitted to our medical facility on August 19, 2021. The patient had a history of hypertension and type 2 diabetes. Upon admission, the patient's blood pressure measured 138/86 mmHg, with a heart rate of 84 beats per minute and regular rhythm. According to the Medical Research Council grading system, physical examination revealed a shallow left nasolabial sulcus, left‐sided tongue deviation, and grade 3 muscle strength in the left limb. In contrast, the right limb displayed normal muscle strength. Muscle tension was normal (tendon reflex++), with a positive Babinski sign on the left and negative on the right. No abnormalities were observed in the superficial and deep sensory systems. The NIHSS score was 7. On day 2 post‐admission, brain magnetic resonance imaging (MRI) revealed diffusion restriction in the right pontine region near the midline (Figure 1A,B); however, the MCPs appeared normal on T1‐ and T2‐weighted images (Figure 1D–F). Computed tomography angiography (CTA) demonstrated multifocal stenosis in the right vertebral and basilar arteries (Figure 1C). Routine blood tests, liver and renal function tests, fibrinogen levels, D‐dimer levels, hypersensitive C‐reactive protein levels, homocysteine levels, total cholesterol levels, and triglyceride levels were within the normal ranges. His fasting glucose was 5.09 mmol/L, and glycosylated hemoglobin was 7.5%.

**FIGURE 2:**
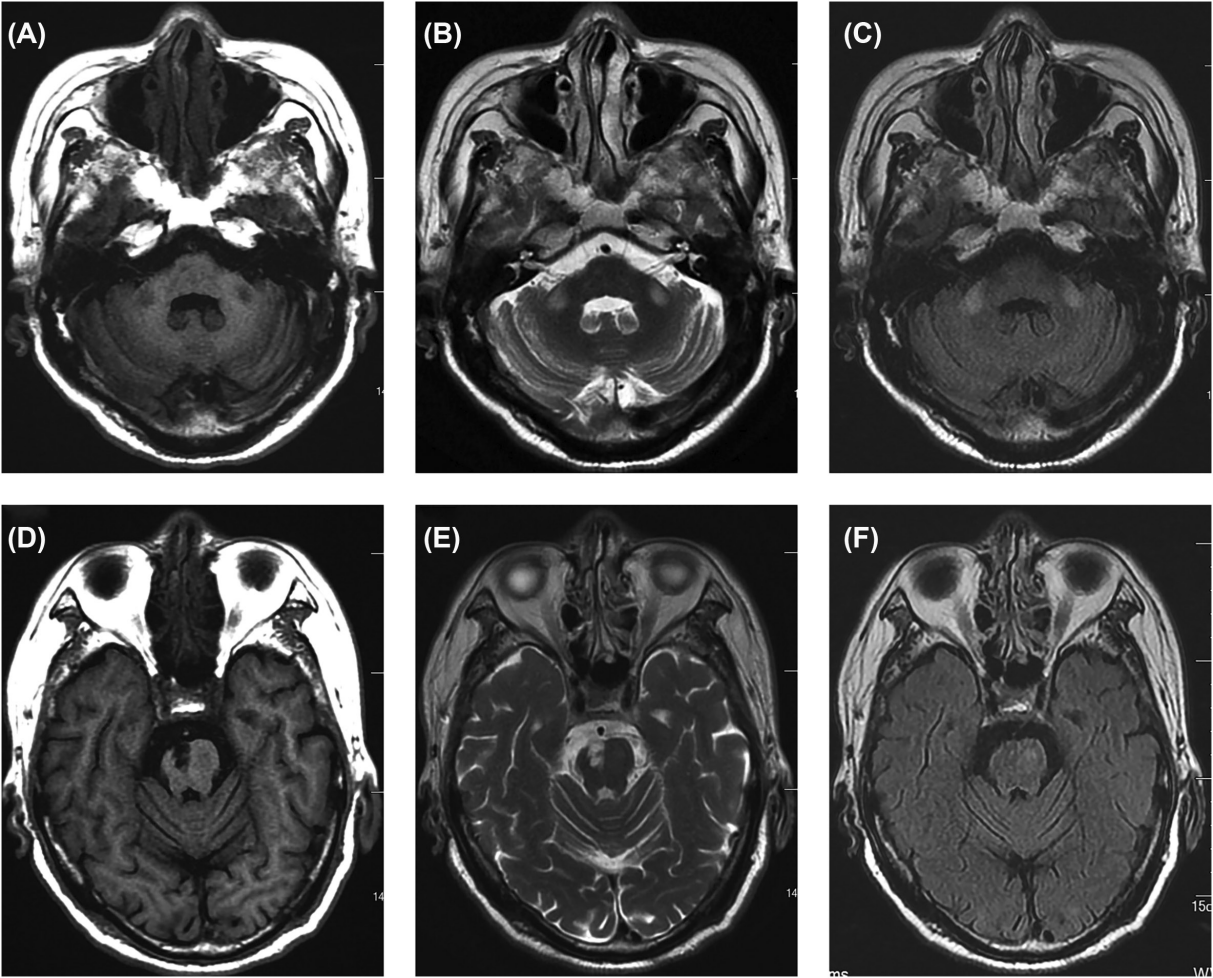
Bilateral symmetrical wallerian degeneration in the middle cerebellar peduncles was observed through MRI. (A–C) symmetrically low T1‐weighted (Axial T1W) signal in the bilateral middle cerebellar peduncles, accompanied by high T2‐weighted (Axial T2W) and fluid‐attenuated inversion recovery (Axial FLAIR) signal intensities. (D–F) low T1W signal intensities in the right pons paramedian, along with high T2W and FLAIR signal intensities, consistent with gliosis/chronic ischemia.

The patient was treated with antiplatelet drugs and statins, and managed for related risk factors. He was discharged with an NIHSS score of 3 and a Modified Rankin Score (mRS) of 2, reporting significant recovery in limb weakness and speech disorder. Secondary prevention included enteric‐coated aspirin tablets, atorvastatin calcium tablets, oral hypoglycemic drugs, and antihypertensive agents. At the 10‐month follow‐up, further improvement in hemiparesis was observed. Brain MRI at the 10‐month follow‐up indicated abnormal signals in the bilateral middle cerebellar peduncles and gliosis in the right pons (Figure 2). The patient resumed his usual activities approximately 18 months after the onset of symptoms.

#### 2.4.2 Patient 2

**FIGURE 3:**
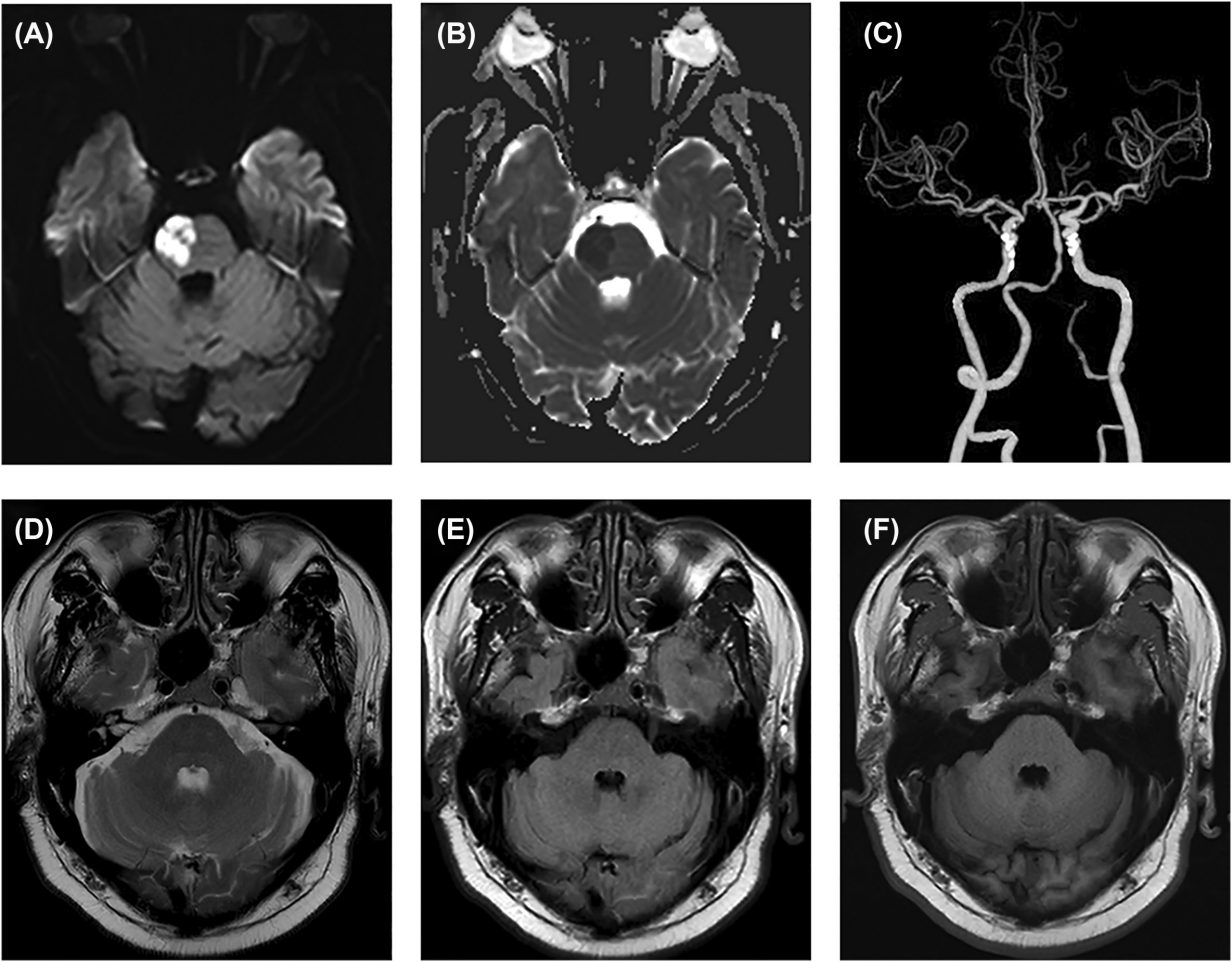
The initial MRI examination of patient 2 revealed acute right pontine infarction. (A, B) High diffusion‐weighted imaging (DWI) signal intensity, and low apparent diffusion coefficient signal intensity, in the right pons. (C) Computed tomography angiography (CTA) showed multifocal stenosis of left vertebral artery and basilar artery. (D–F) There were no abnormal signals in the bilateral middle cerebellar peduncles.

On May 12, 2022, a 62‐year‐old man presented with acute‐onset left limb weakness and was admitted to our medical facility. He had a medical history of long‐standing hypertension, with blood pressure measuring 150/92 mmHg on admission and a heart rate of 74 beats per minute with a regular rhythm. The neurological examination revealed dysarthria, left facial weakness, and muscle weakness in the left limbs. The NIHSS score was 8. On the second day following admission, a brain MRI scan demonstrated restricted diffusion in the right side of the pons, as depicted in Figure 3A,B. The MCPs exhibited no observable alterations in signal intensity on T1‐, T2‐, and T2‐weighted images, as illustrated in Figure 3D–F. Furthermore, CTA of the head and neck showed multifocal stenosis of the left vertebral artery and basilar artery (Figure 3C). Routine blood tests, liver and renal function tests, fibrinogen levels, hypersensitive C‐reactive protein levels, homocysteine levels, total cholesterol levels, triglyceride levels, and low‐density lipoprotein cholesterol (LDL‐C) levels were all within normal ranges. His fasting glucose was 10.25 mmol/L, and glycosylated hemoglobin was 6.5%.

**FIGURE 4:**
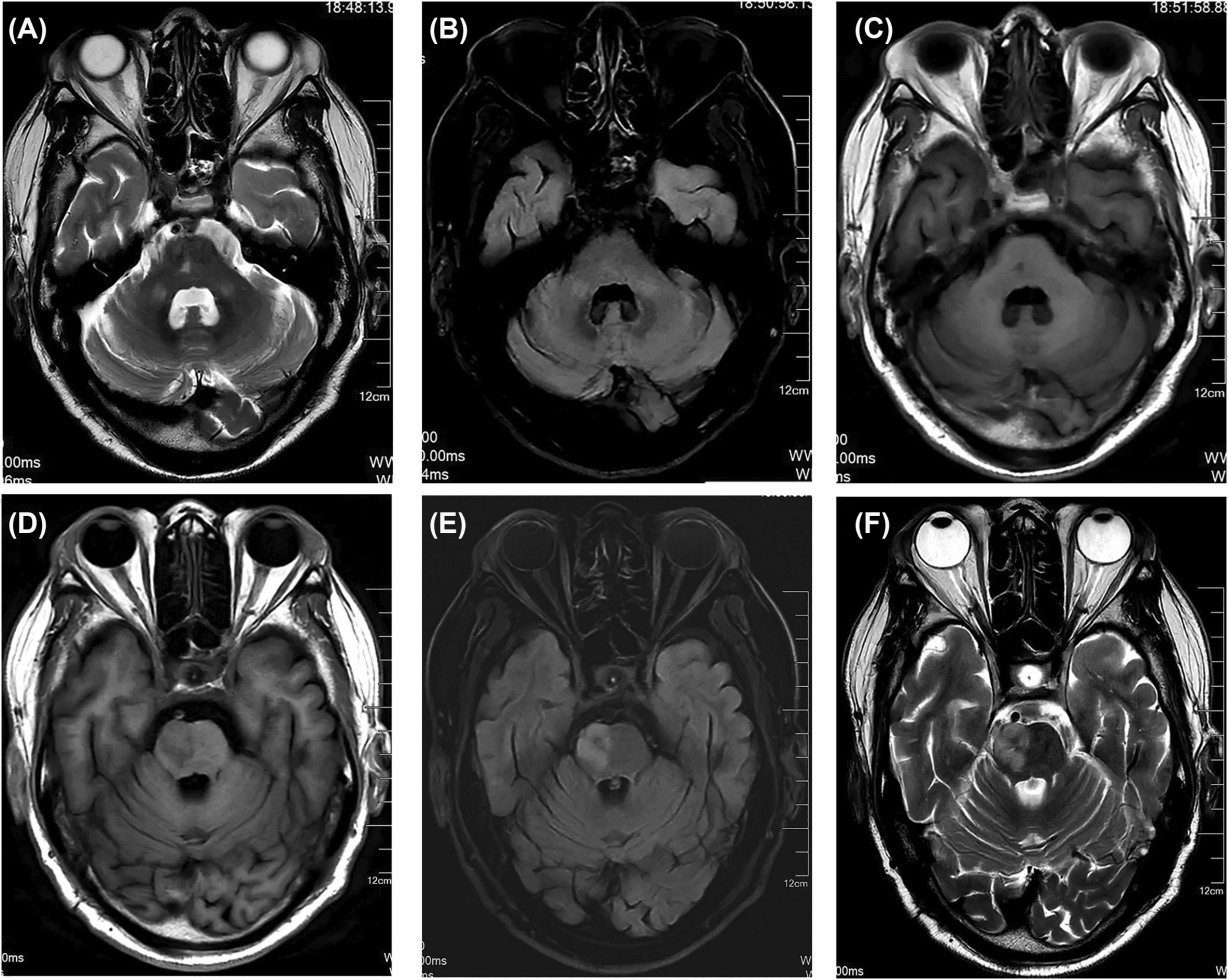
MRI imaging showed bilateral symmetrical wallerian degeneration in the middle cerebellar peduncles. (A–C) Symmetrically hyperintensity on T2/FLAIR WI, with hypointensity on T1 WI in the bilateral middle cerebellar peduncles. Low T1W signal intensities in the right pons, with hyperintensity on FLAIR and T2 WI. (D–F) Low T1W signal intensities in the right pons, with high T2W and FLAIR signal intensities.

He was treated with antiplatelet drugs and statins, and managed for related risk factors. On the tenth day, the patient was discharged with significant improvement in limb weakness and speech disorder, as indicated by an NIHSS score of 3 and a mRS of 2. Secondary prevention included oral hypoglycemic drugs, antihypertensive agents, enteric‐coated aspirin tablets, and atorvastatin calcium tablets. At the 1‐month follow‐up, the MRI revealed a prior pontine infarction and symmetrical hyperintensity in bilateral MCPs on T2‐weighted and fluid‐attenuated inversion recovery (FLAIR) images, suggesting MCP degeneration (Figure 4). After 12 months, the patient returned for reexamination. His unstable feelings were significantly alleviated, whereas neurological examination still revealed slight weakness in the left limbs. Meanwhile, the mRS score decreased to 1.

#### 2.4.3 Patient 3

**FIGURE 5:**
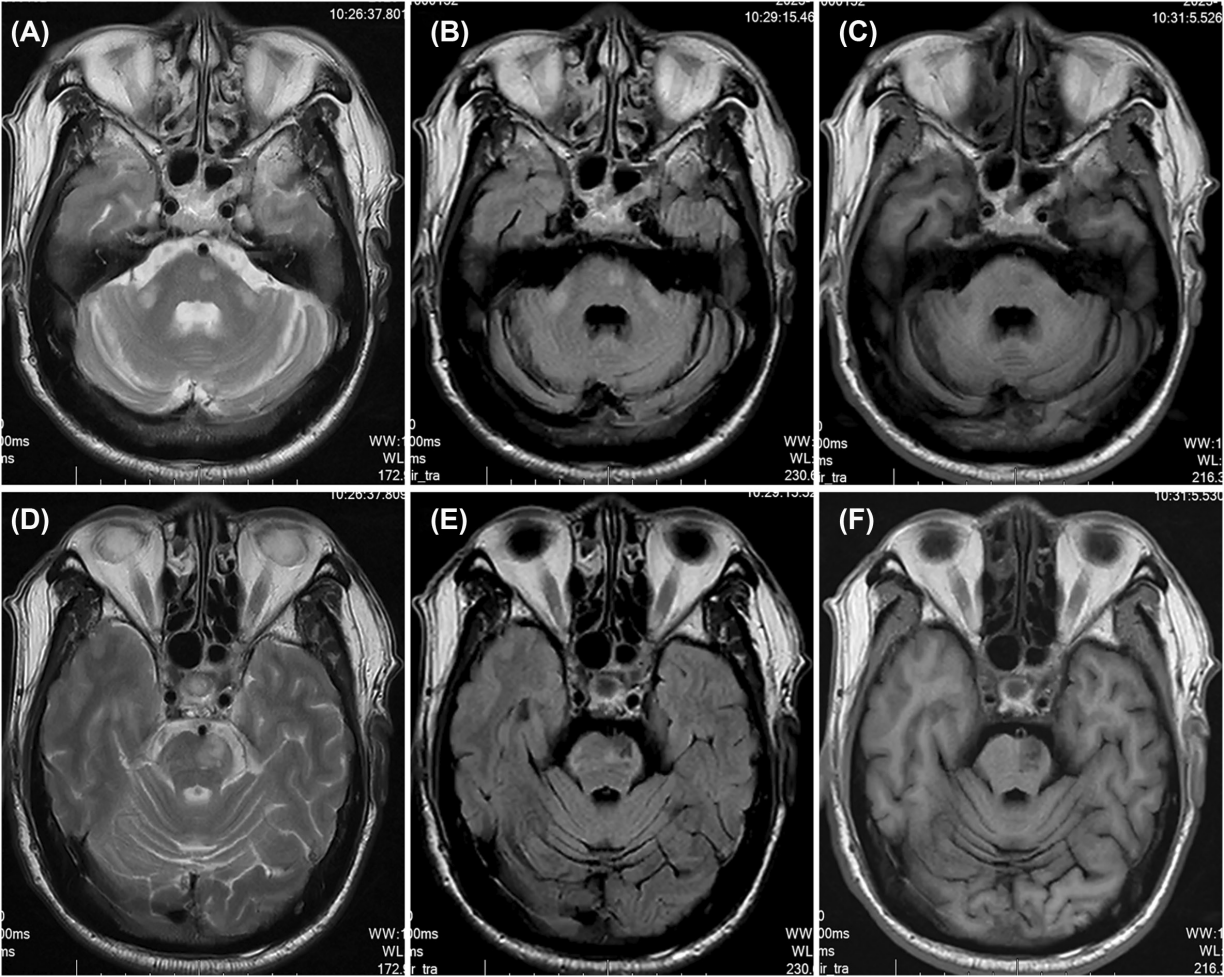
MRI showed bilateral symmetrical wallerian degeneration in middle cerebellar peduncles (patient 3). Symmetrically high T2‐weighted (A) and fluid‐attenuated inversion recovery imaging (B) signal intensities, with low T1‐weighted (C) signal in the bilateral peduncles. Low T1W (D) signal intensities in the left pons, with high T2W (E) and FLAIR (F) signal intensities.

A 73‐year‐old man with a history of smoking and long‐standing hypertension experienced a pontine infarction 10 months before his follow‐up examination. The patient showed good clinical outcomes during follow‐up and did not report any sudden neurological symptoms. Cranial MRI at the 10‐month follow‐up indicated a previous left pontine infarction and symmetrical bilateral MCP hypointensities on T1‐weighted imaging and hyperintensities on T2‐weighted and fluid‐attenuated inversion recovery imaging (Figure 5). When evaluated 18 months after the original onset of symptoms, he had resumed his usual level of activities.

## 3 RESULTS

### 3.1 Clinical presentation

**FIGURE 6:**
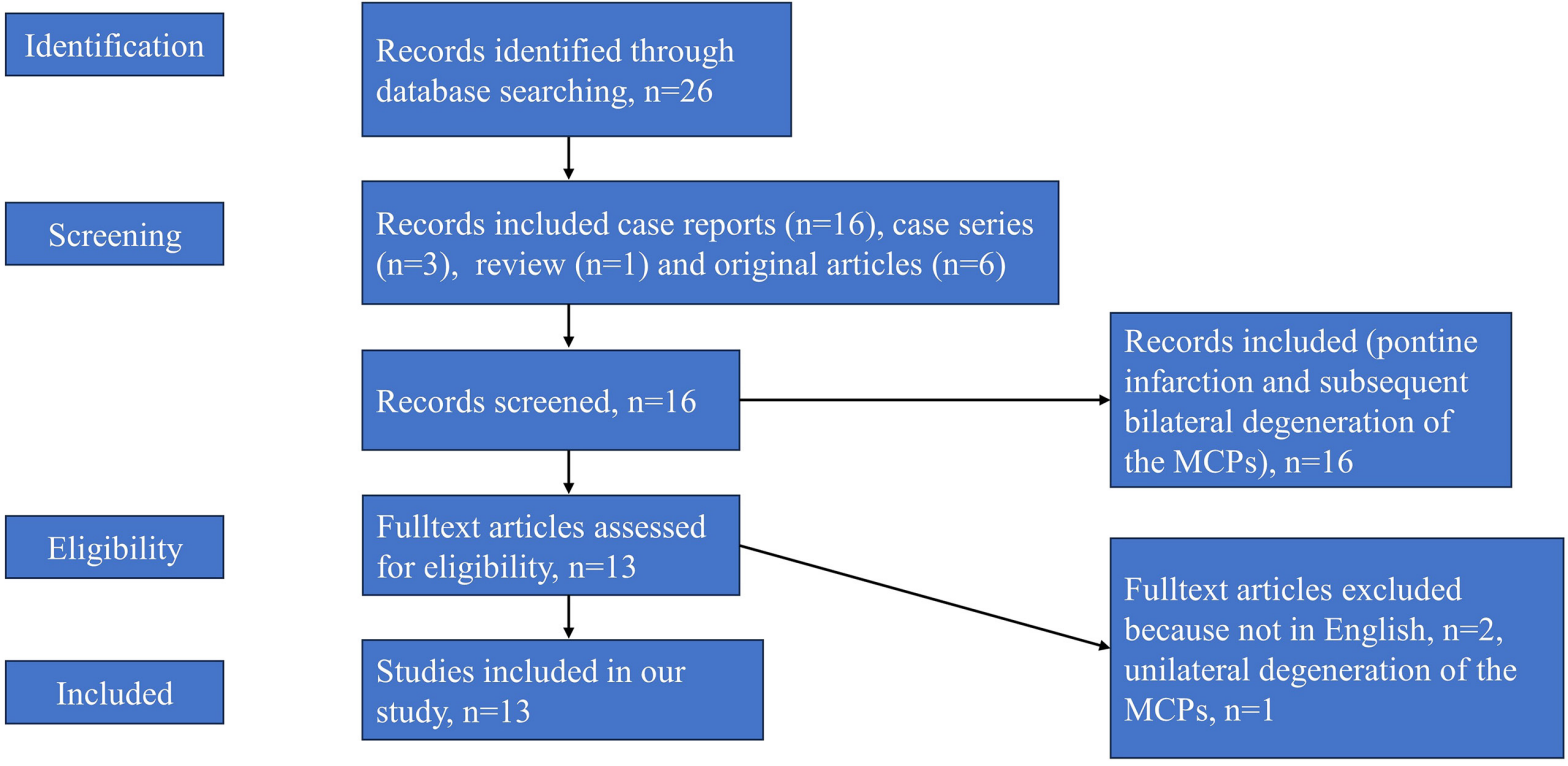
Flow chart detailing the search protocol and the results after application of inclusion and exclusion criteria.

Our literature search identified 26 papers comprising 16 case reports, three case series, one review, and six original articles. The inclusion criteria for our analysis were limited to patients with acute pontine infarction and subsequent bilateral degeneration of the MCPs. Two non‐English studies and one study involving unilateral MCP degeneration were excluded. Consequently, our final analysis included 13 articles, encompassing 22 patients. Adding three patients to the dataset, the total cases reached 25 (Figure 6). Tables 1 and 2 provide a clinical and radiological summary of the cases examined in this study. Of the 25 cases, 18 were men, and seven were women, ranging from 29 to 77 years (mean age: 66.2 years). Paramedian pontine infarcts were observed in all cases, with 14 occurring on the left, eight on the right, and three bilaterally. WD of the MCPs was detected between 21 days and 12 months after pontine infarction onset. Risk factors for cerebrovascular disease were documented in 18 cases, with 17 (94%) exhibiting apparent risk factors, predominantly hypertension (*n* = 14), diabetes mellitus (*n* = 7), hyperlipidemia (*n* = 4), smoking (*n* = 4), drinking (*n* = 1), and migraine (*n* = 1), among which hypertension was the most prevalent. All patients initially presented clinical symptoms of pontine infarction, including hemiparesis, dysarthria, gait ataxia, vertigo, confusion, collapse, bilateral weakness, drowsiness, and facial palsy. A detailed neurological examination, including a comprehensive cerebellar function assessment, was conducted upon WD detection. Of the cases, 20 (80%) did not exhibit any new neurological symptoms, while the remaining five experienced exacerbation of their original symptoms. One case demonstrated a new acute infarct on the anterior right side of the pons, resulting in exacerbated neurological symptoms in four cases (16%) due to WD.

**TABLE 1:** Summary of the clinical presentations of 25 cases.

Reference	Age, years	Sex	Cardiovascular risk factor	Symptom on admission	NIHSS score	Discharge outcome
[6]	71	M	Hypertension, smoking	Vertigo, dysarthria, hemiparesis	5	mRS = 2
[7]	73	M	Hypertension, diabetes mellitus	Hemiparesis, confusion, vertigo, dysarthria	12	mRS = 4
[7]	77	M	Hypertension, hyperlipidemia	Hemiparesis, vertigo, dysarthria	7	mRS = 2
[7]	77	M	Hypertension, smoking, drinking	Hemiparesis, gait ataxia, dysarthria	8	mRS = 2
[7]	68	F	Hypertension	Gait ataxia, dysarthria	4	mRS = 1
[7]	60	M	Hypertension, diabetes mellitus	Hemiparesis, dysarthria	6	mRS = 2
[7]	72	F	Hypertension, hyperlipidemia	Hemiparesis, vertigo, dysarthria	7	mRS = 2
[7]	50	M	Hypertension, hyperlipidemia, smoking	Hemiparesis, dysarthria	6	mRS = 2
[2]	37	F	Migraine	Headache, collapse	N/A	Respiratory failure from a chest infection
[8]	73	M	Hypertension, diabetes	Dysarthria, both‐sided weakness	13	Speaking function improved moderately and could stand without help
[9]	59	M	Hypertension, hypercholesterolemia, diabetes mellitus	Dysarthria, diplopia, hemiparesis	8	NISS = 5
[10]	61	M	N/A	Hemiparesis	N/A	N/A
[11]	66	M	Diabetes mellitus	Hemiparesis, dysarthria	N/A	NISS = 1, mRS = 0
[12]	59	F	N/A	Gait ataxia, dysarthria, hemiplegia	N/A	Mild gait disturbance, slight hemiparesis
[12]	62	F	N/A	Gait ataxia, dysarthria, hemiplegia	N/A	Mild gait disturbance and slight hemiparesis
[12]	73	M	N/A	Gait ataxia, dysarthria, hemiplegia	N/A	Mild gait disturbance, slight hemiparesis
[13]	70	F	N/A	Dysarthria, gait ataxia	N/A	Good clinal improvement
[14]	66	M	N/A	Vertigo, dysarthria, hemiparesis	N/A	Slightly improved
[15]	77	M	Smoking	Dysarthria, hemiparesis, drowsiness	N/A	Discharged with right hemiparesis
[15]	61	M	Hypertension, diabetes mellitus	Diplopia, dysarthria, hemiparesis	N/A	N/A
[16]	63	M	N/A	Facial palsy, hemiparesis, gait ataxia	N/A	N/A
[17]	73	F	No	Headache, vertigo, dysarthria, hemiparesis	N/A	N/A
Our patient 1	72	M	Hypertension, diabetes mellitus	Dysarthria, hemiparesis	7	NISS = 3, mRS = 1
Our patient 2	62	M	Hypertension	Hemiparesis	8	NISS = 3, mRS = 2
Our patient 3	73	M	Hypertension	Hemiparesis	N/A	mRS = 2

Abbreviations: M, male; F, female; mRS, Modified Rankin Score; N/A, not available; NIHSS, National Institutes of Health Stroke Scale.

**TABLE 2:** Summary of the radiological features of 25 cases.

Reference	Age, years	Sex	Pontine infarction site	MRI examination	Follow‐up time	Neurological symptoms at Wallerian degeneration
[6]	71	M	Left	High T2W and FLAIR signal intensity	3 months	No
[7]	73	M	Bilateral	High T2W and FLAIR signal intensity	5 months	No
[7]	77	M	Left	High T2W and FLAIR signal intensity	4 months	No
[7]	77	M	Left	High T2W and FLAIR signal intensity	3 months	No
[7]	68	F	Right	High T2W and FLAIR signal intensity	7 months	Worsening dysarthria and ataxia
[7]	60	M	Right	High T2W and FLAIR signal intensity	5 months	No
[7]	72	F	Right	High T2W and FLAIR signal intensity	4 months	No
[7]	50	M	Left	High T2W and FLAIR signal intensity	6 months	No
[2]	37	F	Right	High T2W, DWI signal intensity, low ADC signal intensity	23 days	No
[8]	73	M	Bilateral	High T2W, FLAIR signal intensity	7 months	No
[9]	59	M	Bilateral	High T2W, DWI signal intensity, low ADC signal intensity	21 days	Diminished facial sensation in trigeminal distribution, dysmetria, dysdiadochokinesia of the lower left extremity
[10]	61	M	Left	High T2W signal intensity	6 weeks	No
[11]	66	M	Left	High T2W, FLAIR, DWI signal intensity	6 months	No
[12]	59	F	Left	High DWI, T2W signal intensity	4 months	No
[12]	62	F	Left	High DWI, T2W signal intensity	4 months	No
[12]	73	M	Left	High DWI, T2W signal intensity	4 months	No
[13]	70	F	Left	High T2W signal intensity	4 months	No
[14]	66	M	Right	High T2W signal intensity	3 months	No
[15]	77	M	Left	High T2W signal intensity	7 months	Transient worsening of right limb weakness
[15]	61	M	Right	High T2W signal intensity	12 months	Acute postural vertigo, left limb weakness
[16]	63	M	Left	High T2W signal intensity	29 days	No
[17]	73	F	Left	High T2W and DWI signal intensity	3 months	Original symptom aggravation (new acute infarct)
Our patient 1	72	M	Right	High T2W, FLAIR signal intensity, low T1W signal intensity	10 months	No
Our patient 2	62	M	Right	High T2W, FLAIR signal intensity, low T1W signal intensity	1 month	No
Our patient 3	73	M	Left	High T2W, FLAIR signal intensity, low T1W signal intensity	10 months	No

Abbreviations: ADC, apparent diffusion coefficient; DWI, diffusion weighted imaging; FLAIR, fluid attenuated inversion recovery; T2W, T2 weighted.

### 3.2 Radiological findings

MRI findings revealed hyperintense T2‐weighted imaging (T2WI) signals, hyperintense FLAIR signals. Restricted diffusion was observed between 21 days and 4 months post‐pontine infarction. In contrast, low ADC signal intensity was detected at 21 days and 23 days post‐infarction, indicating the early stage of WD.

## 4 DISCUSSION

WD is characterized by progressive axonal degeneration and concurrent demyelination, typically following injury to the proximal axon or cell body. While frequently observed as a secondary effect of ischemic stroke, WD can arise from various other conditions, including hemorrhage, neoplasms, surgical procedures, epilepsy, and white matter diseases.^18^

Dynamic MRI alterations categorize WD in the corticospinal tracts into four stages.^19^ The initial stage, within 4 weeks post‐acute infarction, may not exhibit abnormalities in signal intensity on conventional MRI. However, DWI may reveal pronounced signal intensity. Two cases in our literature review were identified in this stage, showing hyperintensity on DWI signal and hypointensity on ADC map. In the second stage, four to 14 weeks post‐infarction, heightened signal intensity is evident on T2WI. After 14 weeks, the third stage sees a notable increase in T2WI signal intensity. Subsequently, the final stage, occurring several years post‐onset, witnesses white matter tract shrinkage accompanied by significant volume loss. Detection of WD in bilateral MCPs typically occurs incidentally during stages 2 and 3 based on dynamic MRI changes.

Symmetrical bilateral MCP hypointensities on T1WI and hyperintensities on T2WI can be observed in various conditions, including acute cerebral infarction, WD, multiple system atrophy, neuromyelitis optica, and primary central nervous system lymphoma.^5^ WD may manifest with stroke‐like restricted diffusion in clinical settings, cautioning against misdiagnosing it as acute ischemic infarction.^20^ Neurologists must carefully correlate distinct radiological characteristics with medical history to reach an accurate diagnosis. Particularly on MRI, crucial distinguishing features between these conditions include the bilateral and symmetrical lesions commonly observed in WD of MCPs, typically located below the old healed pontine infarcts.^21^ Moreover, similar bilateral MCP abnormal MRI findings may occur in Fragile X premutation^22^ or adult‐onset leukodystrophies.^23^ Increased T2 signal intensity at the MCPs is diagnostic for Fragile X‐associated tremor/ataxia syndrome, a progressive neurological disorder affecting premutation carriers, characterized by tremors, balance issues, and other neurological signs. This condition should be considered in the differential diagnosis of WD of MCPs, mainly when prior documentation of pontine infarction is unclear.

WD does not appear to serve as a marker for an unfavorable outcome. Specifically, in cases of pontine infarction, WD in the MCPs is always asymptomatic and detected incidentally, indicative of a poor prognosis.^4^ Research on the prognosis of WD in the MCPs is limited. Zhang et al. observed that most patients with WD had an mRS score >2 at 90 days post‐discharge, indicating a worse prognosis than the control group.^4^ Liang et al. provided evidence that WD in the MCPs after pontine infarction may impede neurological function recovery.^24^ In our research, 20 patients exhibited no clinical symptoms during subsequent visits, while only four reported worsening pre‐existing symptoms. WD of the MCPs can present with ataxia due to interruption of the pathways connecting the motor cortex and cerebellar hemisphere, impacting functions such as movement planning and coordination.^15^, ^25^

WD of bilateral MCPs can ensue following pontine hemorrhage^26^ or central pontine myelinolysis.^5^ However, published data on the incidence and prevalence of bilateral MCP WD are currently lacking. Zhang et al. analyzed data from three large medical centers over 4 years, reporting a detection rate of 4.9%.^4^ Notably, the WD of MCPs typically exhibits bilateral and symmetrical characteristics. The middle cerebellar peduncle contains the fibers of the cortico‐pontine cerebellar tracts. These fibers originate in the contralateral pontine nuclei. Unilateral pontine lesions interrupt the ipisilateral cortico‐pontine fibers and the crossing contralateral pontine‐cerebellar fibers, which explain bilateral WD of MCPs.^27^ Unilateral and isolated pons infarction may induce secondary WD of bilateral MCPs due to the pontine region's unique anatomical structures involved in fiber decussation. Furthermore, there are reports of WD affecting the unilateral MCP following ipsilateral lower pontine infarction.^28^

In summary, this study provides a comprehensive overview of the clinical and radiological characteristics of WD in bilateral MCPs. The majority of patients remain asymptomatic during follow‐up clinic visits, with only a minor subset experiencing worsened pre‐existing neurological symptoms attributed to WD. MRI effectively detects WD between 21 days and 12 months post‐pontine infarction, revealing bilateral MCP lesions characterized by hyperintensity on T2WI and FLAIR. However, it is essential to acknowledge the limitations of current research. Firstly, a small number of participants limited our findings. Expanding the sample size through multi‐center collaboration could enhance the reliability of our results. Given the rarity of this condition, meeting recruitment targets was challenging. However, the insights gleaned from our study provide valuable new information for this patient cohort. Secondly, the short‐term follow‐up period constituted another limitation. Given the infrequency of WD in MCPs, our understanding of its comprehensive characteristics remains incomplete. Thirdly, the need for sensitive clinical outcome measures and accurate measurement of pontine infarct size to comprehensively understand the impact of degeneration on prognosis. Consequently, further investigations are warranted to elucidate the prognosis of WD in MCPs, highlighting the necessity for additional studies in this domain. Further investigation is imperative to delineate WD frequency, clinical characteristics, and radiographic manifestations in bilateral MCPs following pontine infarction. Besides, beyond traditional MRI, advanced imaging modalities can provide more information about MCPs WD, even at earlier stages. These MRI techniques, such as diffusion tensor imaging (DTI), can depict decreased microstructural integrity of both lesional and normal appearing white matter, based on impaired water diffusivity, and considering fractional anisotropy, and radial, and axial diffusivity.^29^ The application of DTI can help identify WD secondary to pons infarction early and measure its impact on neurological function, aiding in enhancing functional recovery.

## FUNDING INFORMATION

This work was supported by National Natural Science Foundation of China (grant no. 81601018), Natural Science Foundation of Shandong Province, China (ZR2021MH043), and Cultivation Fund for the First Affiliated Hospital of Shandong First Medical University (QYPY2021NSFC0617).

## CONFLICT OF INTEREST STATEMENT

The authors have declared that no competing interest exists.

## CONSENT

We have obtained the patient's permission and informed consent for the publishing of her information and images.

## Data Availability

Data available on request from the authors.
